# Data on motivations of food choices obtained by two techniques: Online survey and in-depth one-on-one interview

**DOI:** 10.1016/j.dib.2018.10.108

**Published:** 2018-10-28

**Authors:** Uyen Thuy Xuan Phan, Edgar Chambers

**Affiliations:** Sensory Analysis Center, Kansas State University, 1310 Research Park Dr., Manhattan, KS 66502, United States

**Keywords:** Survey questionnaire, Food choice map, Food choice, Motivation, Eating occasion

## Abstract

This data article provides the data related to the research article entitled “Motivations for meal and snack times: Three approaches reveal similar constructs” (Phan et al., 2018). The data consists of two datasets collected from two research techniques: online survey questionnaire and one-on-one interview. The data include details of the food and beverage items the participants consumed at specific eating occasions together with the motivations associated with consumption of those foods. The data also provides the food groups to which the food items belong, to facilitate different levels of data analysis to explore the relationship between food, eating occasions and people׳s motivations.

**Specifications table**

**Table:** 

Subject area	Food Science
More specific subject area	Food choice
Type of data	Table, Figures
How data was acquired	Online survey questionnaire and one-on-one interview
Data format	Processed
Experimental factors	Participants were people who were older than 18 and have lived in the U.S. for at least 10 years.
Experimental features	The online survey used The Eating Motivation Survey questionnaire. The Food Choice Map technique was used in the one-on-one interview.
Data source location	Manhattan, Kansas, USA
Data accessibility	Data is with this article
Related research article	[1] U.T.X. Phan, E. IV. Chambers. Motivations for meal and snack times: Three approaches reveal similar constructs. Food Qual. Pref. 68 (2018): 267–275

**Value of the data**

• The data provide details of the reasons/motivations people have for choosing a specific dish in their daily diet together with the information about the eating occasion and the food group it belongs to. Therefore, the data can be used to explore the food-eating-motivation relationship at different levels: specific and category, depending on how a researcher wants to look at it.
• Information obtained from the data can be a helpful source of information for any campaign or nutrition program that aims to improve people׳s diet towards healthy eating.
• The research methods presented in this paper provides a new angle to studying food choice as it uses a bottom-up approach in contrast to the traditional top-down method.

## 1 Data

Two different approaches have been used in order to examine the motivations underlying people specific food choices on a daily basis. An online survey targeted a group of 198 people living in the mid-western city in the United States to collect overall reasons for consuming certain foods and beverages for specific eating occasions. One-on-one interviews were then used to extract in-depth information from a group of 100 people about specific motivations for choosing specific foods and beverages in a weekly diet. The data included the name of the food item, the food group it belonged to (classified based on the National Nutrient Database for Standard Reference Release 27 of USDA http://ndb.nal.usda.gov/ndb/foods), the eating occasion at which it was consumed, and the motivations associated with it (1-yes, 0-no). The online survey documented motivations for 477 food and beverage items while the interview collected motivations for 3427 items. The difference in the amount of data between the two approaches were at the fact that in the online survey, each respondent only provided answers for one eating while in the interview, each respondent provided information for all eating throughout the day.

## 2 Experimental design, materials and methods

### 2.1 Online survey

**Fig. 1:**
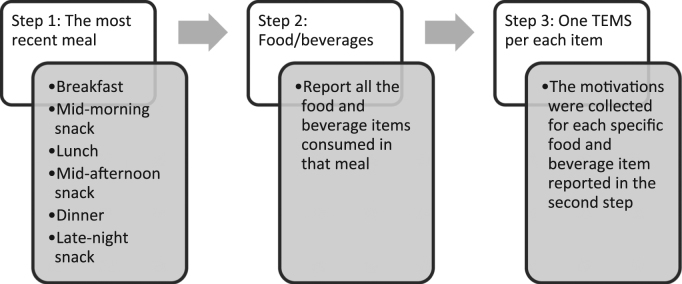
A flowchart of the process the participants went through to answer the online survey questionnaire.

The survey questionnaire included questions about the participants׳ demographics, their most recent eating occasion and which food/beverage items were consumed at that eating occasion. Six eating options were provided: breakfast, mid-morning snack, lunch, mid-afternoon snack, dinner, late-night snack. The questionnaire adapted The Eating Motivation Survey (TEMS) [1], [2], [3], [4], [5] as the main tool to extract the participant׳s motivations for choosing the foods/beverages reported. The adapted TEMS included 17 motivations measured by 3 subscales, for instance, “because I have a an appetite for it”, “because it tastes good”, and “because I like it” were the three subscales for Liking (Table 1). Convenience was measured by 4 subscales and Variety Seeking and Choice limitation were measured by 2 subscales. In total, the adapted TEMS consisted of 50 subscales to cover the 17 motivation constructs (Table 1). Fig. 1 shows the flow of the survey questionnaire. The survey was operated in Qualtrics software (Qualtrics, Provo, UT, USA). The motivation items in TEMS were shown in random order. A Check-All-That-Apply protocol was used due to the large amount of questionnaire items the participants had to handle.

**Table 1:** The 50 subscales used in the eating motivation survey.

Liking	▪ because I have an appetite for it ▪ because it tastes good ▪ because I like it
Habits	▪ because I׳m accustomed to eating it ▪ because I usually eat it ▪ because I am familiar with it
Need and hunger	▪ because I need energy ▪ because it is pleasantly filling ▪ because I׳m hungry
Health	▪ to maintain a balanced diet ▪ because it is healthy ▪ because it keeps me in shape (e.g. energetic, motivated)
Convenience	▪ because it is quick to prepare ▪ because it is the most convenient ▪ because it is easy to prepare ▪ because someone made it for me and it is the choice
Pleasure	▪ because I enjoy it ▪ in order to indulge myself ▪ in order to reward myself
Traditional eating	▪ because it belongs to certain situations ▪ out of traditions (e.g. family traditions, special occasions) ▪ because I grew up with it
Natural concerns	▪ because it is natural (e.g. not genetically modified) ▪ because it contains no harmful substances (e.g. pesticides, pollutants, antibiotics) ▪ because it is organic
Sociability	▪ because it is social ▪ so that I can spend time with other people ▪ because it makes social gatherings more comfortable
Price	▪ because it is inexpensive ▪ because I don׳t want to spend any more money ▪ because it is on sale
Visual Appeal	▪ because the presentation is appealing (e.g. packaging) ▪ because it spontaneously appeals to me (e.g. situated at eye level, appealing colors) ▪ because I recognize it from advertisements or have seen it on TV
Weight control	▪ because it is low in calories ▪ because I watch my weight ▪ because it is low in fat
Affect regulation	▪ because I am sad ▪ because I am frustrated ▪ because I feel lonely
Social norms	▪ because it would be impolite not to eat it ▪ to avoid disappointing someone who is trying to make me happy ▪ because I am supposed to eat it
Social image	▪ because it is trendy ▪ because it makes me look good in front of others ▪ because others like it
Choice limitation	▪ because it was what was served ▪ because it is the only choice
Variety seeking	▪ because I like to eat a variety of different foods each day ▪ because I don׳t like to eat the same food for breakfast everyday

### 2.2 One-on-one interviews

The one-on-one interviews employed the food choice map [6]. The interviewees were first presented with a set of pictures depicting different foods and beverage items in 17 categories [1] such as baked products, fast foods, dairy and egg products, and soup. Interviewees were asked to pick out the food images that represent typical foods and beverages that they often consume in a week. The interviewees used temporary glue to stick the food images onto a large worksheet (0.8 × 1.3 m) in the place that reflected the time of the day and the number of days in a usual week that they ate the food for the same eating occasion (breakfast, mid-morning snack, lunch, mid-afternoon snack, dinner, late-night snack).

**Fig. 2:**
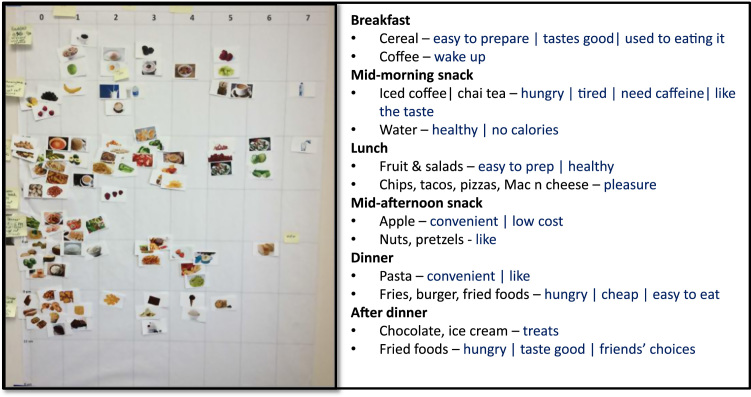
An example of the food choice map and some data collected from the interview based on this map.

After completing the food choice map, the interviewees provided the name for every eating occasion they had on their food map and the reasons (motivations) why they consumed each food or beverage on their map by responding to the question such as “What reasons do you have for choosing … (food/beverage) …for your …(breakfast, lunch, etc.) …?”. All interviews were audio-recorded and note-taken for later data transcription. Fig. 2 shows an example of a food choice map with some data collected from transcribing the interview based on that map.

The survey questionnaires and the interview content were both reviewed and approved by the Internal Review Board of Kansas State University (IRB #7297).
